# Retraction: Effects of LncRNA-HOST2 on cell proliferation, migration, invasion and apoptosis of human hepatocellular carcinoma cell line SMMC-7721

**DOI:** 10.1042/BSR-2016-0532_RET

**Published:** 2021-08-24

**Keywords:** Hepatocellular carcinoma, Human ovarian cancer specific transcript 2, Long noncoding RNA, Proliferation, SMMC-7721 cell line

This Retraction follows an Expression of Concern relating to this article previously published by Portland Press.

This article is being retracted from *Bioscience Reports* at the request of the Editor-in-Chief and the Editorial Board following receipt of a notification from a reader, alerting the Editorial Board to image duplications in Figures 5C and 7A which appear in other publications.

The authors have been contacted in regards to the Retraction, and have not responded to the Journal's queries and the concerns raised. Given the extent of the issues raised, the Editorial Board stand by the decision to retract the article.

